# Evaluation of genotoxic effects of 3-methyl-5-(4-carboxycyclohexylmethyl)-tetrahydro-2H-1,3,5-thiadiazine-2-thione on human peripheral lymphocytes

**DOI:** 10.1080/13880209.2017.1296000

**Published:** 2017-03-05

**Authors:** Ece Avuloğlu-Yılmaz, Deniz Yüzbaşıoğlu, Azime Berna Özçelik, Seyhan Ersan, Fatma Ünal

**Affiliations:** a Faculty of Science, Department of Biology, Genetic Toxicology Laboratory, Gazi University, Ankara, Turkey;; b Faculty of Pharmacy, Department of Pharmaceutical Chemistry, Gazi University, Ankara, Turkey

**Keywords:** Tranexamic acid, genotoxicity, DNA damage, lymhocyte culture

## Abstract

**Context:** Tranexamic acid is commonly used for curing abnormal bleeding in a variety of diseases. In a previous study, 12 different tetrahydro-2H-1,3,5-thiadiazine derivatives were synthesized from the amine group of tranexamic acid. Their antifibrinolytic and antimicrobial activities were compared with tranexamic acid. 3-Methyl-5-(4-carboxycyclohexylmethyl)-tetrahydro-2H-1,3,5-thiadiazine-2-thione (3-MTTT) was the most remarkable one, which may be used as a drug.

**Objectives:***In vitro* genotoxicity of 3-MTTT was investigated using chromosome aberrations (CAs), sister chromatid exchanges (SCEs), micronucleus (MN) and comet assays.

**Materials and methods:** Various concentrations 0.78, 1.56, 3.13, 6.25, 12.50 and 25.00 μg/mL of 3-MTTT were applied to lymphocytes obtained from two donors for periods of 24 and 48 h. A negative (distilled water), a solvent (2:1 PBS:10% NaOH for cultured lymphocyte, and PBS for isolated lymphocytes) and a positive control (MMC for cultured lymphocytes and H_2_O_2_ for isolated lymphocytes) were also maintained.

**Results:** While this compound did not increase the frequency of abnormal cells and CA/cell ratio compared to negative control (except 48 h, 25 μg/mL), it significantly increased the frequency of SCEs at the four highest concentrations at both treatment periods (except 6.25 μg/mL, 48 h). It significantly decreased the MI in all the concentrations at 24 h (except 0.78 μg/mL) and in the highest three concentrations at 48 h. This compound did not significantly increase the frequency of MN and DNA damage compared to negative control. This compound did not affect the replication and nuclear division index.

**Discussion and conclusion:** Our results demonstrated that this compound does not represent a significant risk at the genetic level in *in vitro* human lymphocytes.

## Introduction

Tranexamic acid is one of the most important drugs with antifibrinolytic activity, which is applied orally, intravenously and by infusion (but not used topically). Tranexamic acid is an inhibitor of fibrinolysis and an activator of plasminogen (Wang et al. 2015). It is commonly used for treating abnormal bleeding in a variety of diseases (Ashfaq et al. 2015). The antifibrinolytic effect of tranexamic acid results from the formation of a reversible complex of the drug with plasminogen. Normally, plasminogen binds to fibrin at a lysine binding site and is converted in the presence of tissue plasminogen activator to plasmin. Tranexamic acid blocks the lysine binding site and prevents access of plasminogen to the fibrin molecule (Dunn & Goa 1999). In a previous study, 12 tetrahydro-2H-1,3,5-thiadiazine derivatives were synthesized by Özçelik et al. (2007) from the amine group of tranexamic acid as a prodrug in order to use tetrahydrothiadiazine derivatives topically as bleeding blocking and antimicrobial agents against infections. The antifibrinolytic and antimicrobial activities of these compounds were investigated *in vitro* and compared to tranexamic acid. 3-MTTT was determined as the most prominent compound in that study (Özçelik et al. 2007). Therefore, this compound may be used as a topical drug for blocking bleeding and also preventing infections.

There are some compulsory tests before a drug can be commercialized for human use. One of them is a genotoxicity test. Therefore, in the development of a drug, investigation of the genotoxic potential of a compound as early as possible plays a key role in using or developing that compound in different forms because genotoxicity investigations are generally not performed during the clinical phase (Brambilla & Martelli 2006; Brambilla et al. 2012). Genotoxicity and carcinogenicity studies of tranexamic acid have given negative results. In addition, these studies were performed by the Food and Drug Administration (FDA) and there is no other genotoxicity study of tranexamic acid (Brambilla & Martelli 2009; Brambilla et al. 2012; FDA 2013).

In this study, chromosome aberration (CA), sister chromatid exchange (SCE), micronucleus (MN) and comet tests were applied using The Organization for Economic Co-operation and Development (OECD) guidelines. These test systems are compulsory prior to commercializing a drug for human use and are frequently used as sensitive assays to evaluate the potential genotoxicity, clastogenicity and carcinogenicity of some chemicals (Blasczyk et al. 2003; Yüzbaşıoğlu et al. 2006; Zengin et al. 2011; Doak et al. 2012).

The aim of this study was to evaluate potential genotoxic effects of 3-MTTT using *in vitro* CA, SCE, MN and comet tests in human peripheral lymphocytes. There are no genotoxicity studies about this compound. This study is the first genotoxicity study in human peripheral lymphocytes. Therefore, it will provide important contributions to the literature.

## Materials and methods

**Figure 1.**
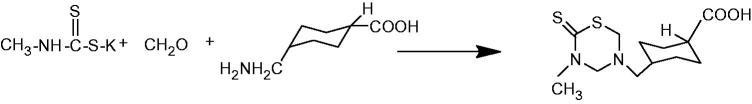
Synthesis of the test substance 3-methyl-5-(4-carboxycyclohexylmethyl)-tetrahydro-2H-1,3,5-thiadiazine-2-thione (Özçelik et al. 2007).

Test substance 3-methyl-5-(4-carboxycyclohexylmethyl)-tetrahydro-2H-1,3,5-thiadiazine-2-thione (Figure 1) was synthesized by Özçelik et al. (2007). 3-MTTT was dissolved in PBS. The other chemicals which are mitomycin C (CAS No: 200-008-6), bromodeoxyuridine (CAS No: 59-14-3), cytochalasin B (CAS No: 14930-96-2), NaCl (CAS No: 7647-14-5) were obtained from Sigma (Sigma-Aldrich Chemie GmbH, Steinheim, Germany). EDTA (CAS No: 6381-92-6), Tris (CAS No: 77-86-1), Triton X-100 (CAS No: 9002-93-1), EtBr (CAS No: 1239-48-8), NaOH (CAS No: 1310-73-2), DMSO (CAS No: 67-68-5), Normal Melting Agarose (CAS No: 9012-36-6), Low Melting Agarose (CAS No: 9012-36-6) were obtained from Applichem (Germany).

Peripheral venous blood was collected from healthy nonsmoking adults, one male and one female aged 27 years. This study was approved by the ethical committee of Gazi University, Faculty of Medicine (15.03.2013 – 68). This study is performed according to the Declaration of Helsinki and informed consent form was obtained from donors. Whole blood was cultured in chromosome medium B (Biochrome (Biochrome GmbH, Berlin, Germany), 2500 μL in each tube) and the cultures were incubated at 37 °C for 72 h. 3-MTTT was added after 24 and 48 h of culture initiation, and colchicine (0.06 μg/mL) was added to each culture at 2 h before harvesting. Six different concentrations of 3-MTTT (0.78, 1.56, 3.13, 6.25, 12.50 and 25.00 μg/mL) were used. To detect the concentrations of 3-MTTT in our tests, LD_50_ values of tranexamic acid (because 3-MTTT was tranexamic acid derivative) in rodents were evaluated and then a preliminary study was carried out to determine the best concentration range compatible with a good cell-proliferating activity, and then resulting in a sufficient number of metaphase for mitotic indices. 3-MTTT exhibited high cytotoxic effect at 50, 100, 200, 400, 800 and 1350 μg/mL concentrations. Also, 3-MTTT decreased the ratio of dividing cells (the mitotic index) at concentrations between 50 and 1350 μg/mL. Therefore, 25 μg/mL was chosen as the highest concentration in this study. In summary, LD_50_ values from rodents of tranexamic acid were used as a reference point. Concentrations used in this study were chosen depending on the values of mitotic index. Additionally, a negative (distilled water), a positive (MMC, 0.20 μg/mL) and a solvent control (2:1 PBS:10% NaOH, final concentration of PBS 4.8 μL/mL) were also used.

The methods of Evans (1984) and Perry and Thompson (1984) were followed in CA and SCE tests with minor modifications (Yüzbaşıoğlu et al. 2006). For the CA test, in total 200 metaphases were examined for each concentration (100 metaphases from each donor). The number of CAs and frequency of abnormal cells were determined. Furthermore, 1000 cells from each donor were examined to determine the MI (totally 2000 cells per concentration). To observe the SCEs, whole blood was cultured in chromosome medium supplemented with 10 μg/mL of bromodeoxyuridine. The slides were stained in Giemsa (5%, pH 6.8) according to Speit and Houpter’s (1985) method, with some modifications (Yüzbaşıoğlu et al. 2006). The SCEs were counted from 50 second mitosis (25 metaphases from each donor) for each concentration. In addition, totally 200 cells were scored to determine the replication index (RI). The replication index (RI) was determined by the formula [RI = M1 + 2(M2) + 3(M3)]/N (total number of scored metaphase (N) and the cells undergoing first (M1), second (M2) and third (M3) divisions) (Schneider et al. 1981).

For the MN test, six different concentrations of 3-MTTT were added after 24 h from the start of the culture. 44 h after initiation of the culture, cytochalasin-B (5.2 μg/mL) was added to block cytokinesis. Slides were made following standard procedure as described by Palus et al. (2003) with some modifications. For each concentration, the MN frequency was assessed by analyzing 1000 binucleated lymphocytes (totally 2000 binucleated cells). Nuclear division index (NDI) was calculated via analysis of 500 cells from each donor (totally 1000 cells) according to the formula [1 × N1] + [2 × N2] + [3 × [N3 + N4]/n where N1–N4 represent the number of cells with 1–4 nuclei (the mono-, bi-, tri-, multi-nucleated cells), and ‘n’ is the total number of cells scored (Surrales et al. 1995).

Comet assay was performed in alkali conditions according to the method of Singh et al. (1988) with some modifications. In order to assess the DNA damage, lymphocytes in blood samples obtained from volunteers were isolated by Biocoll-separating solution directly. The Trypan blue exclusion test was performed to detect the viability of cells. The viability of the cells was higher than 95%. Isolated lymphocytes were incubated for 1 h at 37 °C with six concentrations of the test substance. A negative, a positive (100 mM H_2_O_2_, 0.30 μg/mL) and a solvent control (PBS, 500 μl/mL) were also included. After incubation, the lymphocytes were centrifuged and the supernatant was discarded. The bottom residue was resuspended in PBS and mixed with low-melting point agarose. This suspension was layered onto slides precoated with normal-melting point agarose and covered with a cover slip. The slides were kept at 4 °C for 20 min. Then, coverslips were removed and the slides were put into a cold lysing solution (2.5 M NaCl, 100 mM EDTA, 10 mM Tris pH = 10, in which 10% DMSO and 1% Triton X-100 were added) at 4 °C for at least 1 h. After lysis, slides were placed on an electrophoresis platform filled with an electrophoresis buffer (300 mM NaOH, 1 mM EDTA pH >13). For unwinding of the DNA, slides were left in this buffer for 20 min. Then, DNA was electrophoresed for 20 min at 25 V, 300 mA to detect primary DNA damaging effect caused by the test substance. Slides were then rinsed three times with a neutralization buffer. Each slide was stained with ethidium bromide and analyzed using specialized image analysis system (Comet Assay IV, Perceptive Instruments Ltd., UK). Two slides were prepared for each concentration of 3-MTTT. The tail length (μm), tail intensity (%) and tail moment of the randomly selected 200 cells (100 cells from each of the two replicate slides) were determined.

The obtained data were analyzed using *z* and *t* tests. The *z*-test was used for the percentage of abnormal cells with CA, CA/cell, RI, NDI, MI and MN, and the *t* test was used for SCEs and comet assays. Concentration–response relationships were identified from the correlation and regression coefficients for the percentage of abnormal cells, CA/cell, SCE and MI.

## Results

According to the CA test results, 24 h treatment of 3-MTTT did not increase the frequency of chromosomal aberrations compared with negative and solvent control. In contrast, 48 h treatment of 3-MTTT increased the frequency of CAs, but this increase was not significant compared with negative and solvent controls (except 25 μg/mL) (Table 1).

**Table 1.** Chromosome aberrations induced by 3-MTTT in cultured human lymphocytes.

	Treatment									
	Period	Number of	Concentration	Aberrations						
Test substance	(hour)	metaphases analyzed	(μg/mL)	ctb	csb	scu	f	dic	Abnormal cell ± SE (%)	CA/cell ± SE
Negative control	24	200	0.00	5	2	2	–	1	5.00 ± 1.54	0.050 ± 0.015
Solvent control	24	200	PBS + NaOH	5	–	3	–	1	4.50 ± 1.47	0.045 ± 0.015
MMC	24	200	0.20	16	1	5	–	3	12.50 ± 2.34^a1,b2^	0.125 ± 0.023^a1,b2^
3-MTTT	24	200	0.78	4	–	1	1	–	3.00 ± 1.21	0.030 ± 0.012
		200	1.56	4	3	1	–	–	4.00 ± 1.39	0.040 ± 0.014
		200	3.13	8	2	–	–	–	5.00 ± 1.54	0.050 ± 0.015
		200	6.25	5	2	1	1	1	5.00 ± 1.54	0.050 ± 0.015
		200	12.50	10	1	–	–	–	5.50 ± 1.66	0.055 ± 0.016
		200	25.00	12	1	–	1	–	7.00 ± 3.38	0.070 ± 0.018
Negative control	48	200	0.00	9	–	1	–	–	4.50 ± 1.47	0.050 ± 0.015
Solvent control	48	200	PBS + NaOH	8	2	1	–	–	5.50 ± 1.61	0.055 ± 0.016
MMC	48	200	0.20	29	8	11	–	2	23.50 ± 3.00^a1,b2^	0.250 ± 0.031^a1,b2^
3-MTTT	48	200	0.78	3	–	1	–	2	3.00 ± 1.21	0.030 ± 0.012
		200	1.56	5	1	3	–	1	5.00 ± 1.54	0.050 ± 0.015
		200	3.13	8	2	1	1	2	7.00 ± 1.80	0.070 ± 0.018
		200	6.25	4	3	1	1	1	5.00 ± 1.54	0.050 ± 0.015
		200	12.50	11	4	1	1	–	8.50 ± 1.97	0.085 ± 0.020
		200	25.00	33	3	1	–	–	14.00 ± 2.45^a1,b1^	0.185 ± 0.027^a1,b2^
Frequency of abnormalities (%)				66.79	13.06	12.69	2.24	5.22		

ctb: Chromatid break, csb: chromosome break, scu: sister chromatid union, f: fragment, dic: dicentric chromosome.

a1 Significantly different from the control *p* < 0.001 (*z* test).

b1 Significantly different from the solvent control *p* < 0.01 (*z* test).

b2 Significantly different from the solvent control *p* < 0.001 (*z* test).

The results of the SCE assay in human lymphocyte are presented in Table 2. This compound significantly increased the SCE/cell ratio at four of the highest concentrations at both 24 h and 48 h periods compared with the negative control (except 6.25 μg/mL for 48 h). In addition, compared with the solvent control, this increase was significant at the four highest concentrations for 24 h treatment but at the two highest concentrations for 48 h treatment.

**Table 2.** Effects of 3-MTTT on SCE, RI, and MI in cultured human lymphocytes.

	Treatment									
Test substance	Period (hour)	Number of metaphases analyzed for SCE	Concentration (μg/mL)	Min–max SCE	SCE/cell ± SE	M_1_	M_2_	M_3_	RI ± SE	MI ± SE
Negative control	24	50	0.00	1–8	3.22 ± 0.21	55	54	91	2.18 ± 0.08	9.35 ± 0.65
Solvent control	24	50	PBS + NaOH	1–7	3.20 ± 0.21	50	78	72	2.11 ± 0.08	8.85 ± 0.64
MMC	24	50	0.20	12–34	12.06 ± 0,82^+¡^	80	37	83	2.02 ± 0.07	5.75 ± 0.52^a3,b2^
3-MTTT	24	50	0.78	1–5	2.88 ± 0.15	75	62	63	1.94 ± 0.07	7.85 ± 0.60
		50	1.56	2–6	3.68 ± 0.18	62	51	87	2.13 ± 0.08	7.45 ± 0.59^a1^
		50	3.13	2–6	4.02 ± 0.14^+¡^	87	44	69	1.91 ± 0.07	6.95 ± 0.57^a2,b1^
		50	6.25	3–8	5.08 ± 0.19^+¡^	108	49	43	1.68 ± 0.05	6.70 ± 0.56^a2,b1^
		50	12.50	4–11	6.26 ± 0.22^+¡^	122	56	22	1.50 ± 0.04	6.60 ± 0.56^a2,b1^
		50	25.00	4–15	7.58 ± 0.29^+¡^	118	53	29	1.56 ± 0.04	4.85 ± 0.48^a3,b2^
Negative control	48	50	0.00	2–7	3.76 ± 0.20	51	54	95	2.22 ± 0.09	7.35 ± 0.58
Solvent control	48	50	PBS + NaOH	2–9	4.30 ± 0.18	58	44	98	2.20 ± 0.09	6.80 ± 0.56
MMC	48	50	0.20	14–32	22.98 ± 0.86^+¡^	92	50	58	1.83 ± 0.06	5.00 ± 0.49^a3,b2^
3-MTTT	48	50	0.78	1–6	3.10 ± 0.16	72	58	70	1.29 ± 0.02	7.30 ± 0.58
		50	1.56	1–9	3.70 ± 0.25	43	52	105	2.31 ± 0.09	7.00 ± 0.57
		50	3.13	2–9	5.02 ± 0.28^+^	58	66	76	2.09 ± 0,08	6,70 ± 0.56
		50	6.25	2–9	4.32 ± 0.25	65	60	75	1.95 ± 0.67	5.80 ± 0.52^a1^
		50	12.50	3–13	6.30 ± 0.31^+¡^	168	29	3	1.18 ± 0.01	5.30 ± 0.50^a2^
		50	25.00	4–13	6.24 ± 0.28^+¡^	169	17	14	1.23 ± 0.02	3.75 ± 0.42^a3,b2^

+ Significantly different from the control *p* < 0.05 (*t*-test).

¡ Significantly different from the solvent control *p* < 0.05 (*t*-test).

a1 Significantly different from the control *p* < 0.05 (*z* test).

a2 Significantly different from the control *p* < 0.01 (*z* test).

a3 Significantly different from the control *p* < 0.001 (*z* test).

b1 Significantly different from the solvent control *p* < 0.05 (*z* test).

b2 Significantly different from the solvent control *p* < 0.001 (*z* test).

The effect of 3-MTTT on the MI was determined and a statistically significant difference was observed between treatment and control cultures (Table 2). This compound decreased the MI in all the concentrations compared with the negative and solvent controls at 24 h treatment period (except 0.78 μg/mL compared to negative control; except 0.78 and 1.56 μg/mL compared to solvent control). In addition, MI decreased in three of the highest concentrations of test substance at 48 h period compared with the negative control and only 25 μg/mL was significant compared to the solvent control. On the other hand, no statistically significant deviation was observed in the RI (Table 2).

Table 3 shows the effects of 3-MTTT on MN frequency and NDI in human peripheral lymphocytes. No significant difference was observed in treatment groups compared with controls, either in MN frequency or in NDI.

**Table 3.** Micronucleus frequency induced by 3-MTTT in cultured human lymphocytes.

	Treatment			Distribution of BN cells according to the number of MN				
Test substance	Period (hour)	Binucleated cells (BN) scored	Concentration (μg/mL)	(1)	(2)	(3)	MN ± SE (%)	Nuclear division index (NDI) ± SE
Negative control	48	2000	0.00	2	–	–	0.10 ± 0.07	1.52 ± 0.39
Solvent control	48	2000	PBS + NaOH	3	–	–	0.15 ± 0.09	1.75 ± 0.42
MMC	48	2000	0.20	22	1	1	1.35 ± 0.26^a1,b1^	1.53 ± 0.39
3-MTTT	48	2000	0.78	2	–	–	0.10 ± 0.07	1.56 ± 0.39
		2000	1.56	3	–	–	0.15 ± 0.09	1.42 ± 0.37
		2000	3.13	3	–	–	0.15 ± 0.09	1.42 ± 0.37
		2000	6.25	3	–	–	0.15 ± 0.09	1.28 ± 0.36
		2000	12.50	4	–	–	0.20 ± 0.01	1.06 ± 0.32
		2000	25.00	3	1	1	0.40 ± 0.14	1.08 ± 0.33

a1 Significantly different from the control *p* < 0.001 (*z* test).

b1 Significantly different from the solvent control *p* < 0.001 (*z* test).

Three different evaluations including tail length, tail intensity and tail moment were performed in the comet assay. According to the comet assay results, test substance did not cause DNA damage at all the concentrations in the mean tail intensity, tail length and tail moment (Table 4).

**Table 4.** DNA damaging effects of 3-MTTT in isolated human lymphocytes.

	Treatment					
Test Substance	Period (hour)	Number of analyzed cells	Concentration (μg/mL)	Mean tail intensity ± SE	Mean tail length ± SE	Mean tail moment ± SE
Negative Control	1	200	0.0	6.04 ± 0.94	48.69 ± 0.87	1.26 ± 0.12
H_2_O_2_	1	200	100 (μM)	13.10 ± 1.35^a^^,^^b^	81.76 ± 3.01^a^^,^^b^	5.02 ± 1.20^a^^,^^b^
Solvent control	1	200	PBS	5.90 ± 0.76	47.57 ± 1.24	1.50 ± 0.17
3-MTTT	1	200	0.78	5.53 ± 0.61	47.75 ± 0.91	1.11 ± 0.13
		200	1.56	7.51 ± 0.81	48.71 ± 0.93	1.64 ± 0.20
		200	3.13	7.12 ± 0.75	49.57 ± 1.20	1.48 ± 0.16
		200	6.25	6.99 ± 0.73	50.50 ± 0.84	1.45 ± 0.15
		200	12.50	8.16 ± 0.80	49.52 ± 0.98	1.75 ± 0.18
		200	25.00	8.61 ± 0.95	50.97 ± 1.23	1.92 ± 0.28

a Significantly different from the control *p* < 0.05 (*t*-test).

b Significantly different from the solvent control *p* < 0.05 (*t*-test).

## Discussion

Tranexamic acid is an antifibrinolytic drug that is applied orally, intravenously and by infusion. However, it has not been used topically. Substituted tetrahydro-2H-1,3,5-thiadiazine-2-thione derivatives are synthesized using the tranexamic acid as a prodrug. It is recognized that substituted tetrahydro-2H-1,3,5-thiadiazine-2-thione derivatives have several biological activities such as antibacterial, antifungal and antiviral. According to Özçelik et al. (2007), 3-MTTT is the most effective one among them (Özçelik et al. 2007). Therefore, in the future, this compound might be used as a kind of injury strip topically. Current guidelines for genotoxicity testing of pharmaceuticals (U.S. Department of Health and Human Services, Food and Drug Administration, Center for Drug Evaluation and Research, 1997a,b) report a standard test battery that consists of a gene mutation test using bacteria, cytogenetic evaluation of chromosomal damage or gene mutation test using *in vitro* mammalian cells and using *in vivo* rodent hematopoietic cells for testing chromosomal damage (Brambilla et al. 2009). 3-MTTT is a new compound and for this reason these tests should be performed. CAs, SCE and MN analysis of human lymphocytes as well as comet assay are used as the most useful tests to detect the potential genotoxicity of chemicals (Blasczyk et al. 2003; Bonassi et al. 2010; Chandirasekar et al. 2014; Santovito et al. 2014). They have been considered to be markers of the early biological effects of carcinogen exposure (Liou et al. 2002).

Chromosomal aberration is one of the main endpoints indicating genotoxicity and mutagenicity of chemicals (Fei et al. 2015). It is known that the increased level of CAs in lymphocytes significantly elevates the risk of developing cancer (Hagmar et al. 1998; Chandirasekar et al. 2014). Good correlation has been found between chromosomal aberration induction and MN induction (Fei et al. 2015). 3-MTTT induced structural CAs only at the highest concentration for 48 h treatment and did not cause a significant increase in MN frequency. Data obtained here demonstrated that 3-MTTT did not induce CA (except for 48 h, 25 μg/mL) and MN formation.

The MN assay that has the capacity to detect both aneugens (chromosome lagging due to dysfunction of mitotic apparatus) and clastogens (chromosome breakage) is widely applicable in different cell types (Kirsch-Volders et al. 2011). Thus, clastogenic and/or aneugenic agents are able to arise chromosomal fragmentation or chromosomal losses during the cellular division that are not integrated in the nucleus of daughter cells, resulting in MNi (Fenech 2000; Araldi et al. 2015). MN assay is an early diagnostic test proven to be highly beneficial to check the progress of precancerous lesions followed by its early treatment (Stich et al. 1982; Jyoti et al. 2015). It was also reported that increased MN frequency is related to cytotoxicity (Kirkland 2010). In this study, MN analysis showed that 3-MTTT did not affect the frequency of micronucleated cells in human lymphocytes. These results show that the test substance is not aneugenic and clastogenic in human lymphocytes.

Another test used in our investigation was the SCE test. 3-MTTT induced the formation of SCEs. SCEs could be induced by S-phase-dependent clastogens and they are formed during the S phase of the cell cycle (Wilson & Thompson 2007; Garcia-Sagredo 2008). However, it was indicated that cancer risk is not predicted by the frequency of SCE (Norppa et al. 2006). The biological importance of increasing SCE rate for mutagenesis and carcinogenesis is unclear (Speit & Henderson 2005). SCE is a short-term test for the detection of reciprocal exchanges of DNA between two sister chromatids, involving DNA breakage and subsequent re-joining that occur at low levels in untreated cells (Genualdo et al. 2015). At the same time, SCE may also increase the frequency of DNA single strand breakage (Wilson & Thompson 2007). In this study increase in SCE frequency seems to be resulted from 3-MTTT treatment, but this effect was not shown by CA and MN tests. 3-MTTT may cause DNA damage by affecting the replication mechanism. However, SCE test is not sensitive enough to show DNA damage alone (Eastmond 2014). Therefore, SCE test should be studied with other complementary tests such as CAs (Bozkurt et al. 2004) as we did in this study.

Comet assay has become one of the most effective, popular and widely used methods and it is quick, highly sensitive and applicable to a number of cells. Comet assay allows detection of breaks in DNA strands, double-strand breaks (DSBs) and single-strand breaks (SSBs). These breaks are associated with CAs and genomic instability. The genomic instability is directly associated with malignancy (Yılmaz et al. 2014; Araldi et al. 2015). 3-MTTT did not cause DNA damage at applied concentrations.

In this study, 3-MTTT had no effect on the RI and NDI; however, it significantly decreased the MI, especially at higher concentrations. RI and NDI are indicators of cell proliferation and its kinetics. MI is used for the determination of cell toxicity and it is assumed as a measure of general cytotoxicity (Eroğlu et al. 2007; Ionescu et al. 2011). In this study, there was a decrease in MI frequency compared to the negative and solvent controls. The decrease of the MI could be due to a slower progression of cells from S (DNA synthesis) phase to M (mitosis) phase of the cell cycle or blocking of G2. It may be caused by a decrease in the ATP level. Test compound exposure caused impairment in cell cycle progression but more experiments are needed to elucidate the biochemical mechanisms. At present, there is not enough knowledge on the MI of the test compound on biological systems (Yüzbaşıoğlu et al. 2006; Patlolla et al. 2015).

As previously indicated, 3-MTTT is a tranexamic acid derivative. Tests on tranexamic acid’s possible genotoxicity were performed by the Food and Drug Administration (FDA) with respect to various *in vitro* and *in vivo* test systems. However, no detailed information was found about the genotoxicity test in the reported results. According to this report, tranexamic acid gave negative results in the Ames test, *in vitro* and *in vivo* cytogenetic test systems (*in vitro* CA test in Chinese hamster cells and in *in vivo* CA tests in mice and rats). In addition, positive carcinogenicity in mice has been identified against tranexamic acid but long-term carcinogenicity studies in rats (except biliary system tumors) indicated a negative response (FDA 2013).

Another antifibrinolytic drug, aminocaproic acid, showed a positive response in Ames test (Lewis & Tarrant 1971; Snyder & Green 2001). In addition, the antifibrinolytic drug aprotinin was abandoned after identification of major side effects (European Medicines Agency 2012). Apart from these, no genotoxicity study was reported on any of antifibrinolytic drugs.

In conclusion, the data presented here reveal that 3-MTTT had no genotoxic effect in three of the four assays. It only induced SCE formation. SCEs are reciprocal exchanges of chromatid segments that occur at low levels in untreated cells (Eastmond 2014). They form during the S phase of the cell cycle and can be induced by chemicals that are S phase-dependent DNA-damaging agents (Johnson et al. 2009). The SCE assay has been widely used to assess the genotoxic potential of mutagenic and carcinogenic agents although it has to be taken into account that SCEs do not predict cancer as well as other biomarkers like CAs (Norppa et al. 2006; Sebastià et al. 2014). In the light of this information, it can be stated that 3-MTTT does not represent an important risk at the genetic level *in vitro*. It should be consider that these results were observed in only selected sample size, concentrations and experimental conditions.

3-MTTT may be used as a drug in the near future which can be used topically to stop bleeding and also to generate antimicrobial effect against infections without damaging or at least causing minimum damage to human health especially to the genetic material. But, this drug should be investigated comprehensively by examination and evaluation phase such as *in vivo* studies. On the other hand, it should be allowed to use specified doses by relevant organizations. This study may be used as a reference for other studies in different types of cells or animals.
